# Differentiating Slowly Progressive Subtype of Lower Limb Onset ALS From Typical ALS Depends on the Time of Disease Progression and Phenotype

**DOI:** 10.3389/fneur.2022.872500

**Published:** 2022-05-18

**Authors:** Huagang Zhang, Lu Chen, Jinzhou Tian, Dongsheng Fan

**Affiliations:** ^1^Department of Neurology, Peking University Third Hospital, Beijing, China; ^2^Beijing Key Laboratory of Biomarker and Translational Research in Neurodegenerative Diseases, Beijing, China; ^3^Neurology Centre, Dongzhimen Hospital, Beijing University of Chinese Medicine, Beijing, China; ^4^Key Laboratory for Neuroscience, National Health Commission/Ministry of Education, Peking University, Beijing, China

**Keywords:** amyotrophic lateral sclerosis, disease progression, survival, lumbosacral region, prognosis

## Abstract

**Background:**

Flail leg syndrome (FLS) is a regional variant of amyotrophic lateral sclerosis (ALS) with the characteristics of slow progression and the symptoms confined to the lumbosacral region for extended periods. However, FLS may not be easily differentiated from typical ALS.

**Objective:**

The objective of the study was to determine a cutoff time of disease progression that could differentiate FLS from the typical lower limb onset ALS.

**Methods:**

A cutoff point analysis was performed with maximally selected log–rank statistics in patients with lower limb onset ALS registered from 2009 to 2013. Based on the cutoff duration from the lower limb onset to second region significantly involved (SRSI), all patients were divided into the slowly progressive subtype of lower limb onset ALS group and the typical lower limb-onset ALS group. Patients with the slowly progressive subtype of the lower limb onset ALS, who had the flail leg phenotype, were classified as patients with FLS. Differences between groups were analyzed.

**Results:**

Among the 196 patients recruited, 157 patients with a duration <14 months from lower limb onset to SRSI were classified as having typical lower limb onset ALS. Twenty-nine patients with a duration more than or equal to 14 months and the flail leg phenotype were classified as having FLS. Patients with FLS exhibited a median diagnostic delay of 25 months, a median duration of 24 months from lower limb onset to SRSI, a forced vital capacity abnormity rate of 12.5% at the first visit to our department, and a median survival time of 80 months, which were significantly different from those of patients with typical lower limb onset ALS (*p* < 0.001, *p* < 0.001, *p* = 0.024, *p* < 0.001). The 5-year survival rate of the FLS group (79.3%) was much higher than that of the other group (1.9%).

**Conclusions:**

A crucial feature in differentiating FLS from typical lower limb onset ALS in Chinese patients may be symptoms confined to the lumbosacral region for at least 14 months, which may be better than 12 or 24 months used in the previous studies. The FLS was characterized by slower progression, less and later respiratory dysfunction, and a more benign prognosis than the typical lower limb onset ALS.

## Introduction

Amyotrophic lateral sclerosis (ALS) with lower limb onset is characterized by an insidious onset of weakness or muscle atrophy in the legs without sensory symptoms. The upper limbs or bulbar region are progressively involved several months after onset, and the median survival time of patients with a typical ALS is 3–6 years (1–3). However, the clinical experience has shown that a small group of patients with lower limb onset ALS progress more slowly than typical ALS, and these patients seem to have prolonged survival. Flail leg syndrome (FLS) is a regional variant of ALS with symptoms confined to the lumbosacral region for extended periods (4). Compared with typical lower limb onset ALS, FLS has the characteristics of slower progression and longer survival time. The mean survival of patients with FLS was reported to be between 75.9 and 87 months (5, 6). Due to the lack of specific treatment for ALS, it is important to distinguish FLS from typical lower limb onset ALS to predict prognosis, which plays a decisive role in the choice of treatment and patient care.

Some common clinical features of FLS have been identified, including progressive distal onset with weakness and decreased or absent deep tendon reflexes in lower limbs (4–8). However, FLS may not be easily differentiated from typical ALS or not even be clearly diagnosed in patients with long survival. The diagnostic criteria of FLS vary among different studies. In two case series, the UK and Melbourne study and the US and major academic center study, patients with FLS were included according to the criterion of the duration of time, for which symptoms were confined to the lumbar region for 12 and 24 months, respectively (5, 6). The inclusion criteria for patients with FLS did not include the time of disease progression in a report of Chiò et al. (8). Other features of FLS were quite different, such as the gender ratio and development pattern of weakness. The cutoff time of 12 or 24 months was defined mainly based on clinical experience, which is still not widely recognized.

The most distinct characteristics of FLS, which are different from those of typical ALS with rapid progression, are clinical symptoms confined to the lumbosacral region for extended periods and a better prognosis with a longer survival rate (4–6). In this study, we investigated the clinical features of patients with lower limb onset ALS, analyzed the correlation between disease progression and survival, and calculated the cutoff time of disease progression that could differentiate the slowly progressive subtype of lower limb onset ALS from typical lower limb onset ALS well. Patients with the slowly progressive subtype of lower limb onset ALS, who had the flail leg phenotype, were classified as patients with FLS. The demographic and clinical characteristics of FLS were further described and compared with those in the previous studies.

## Materials and Methods

### Patients

This study was a prospective cohort study and the data analyzed were from the patients with ALS database of Peking University Third Hospital (PUTH). We reviewed the medical records of all patients with a clinical diagnosis of lower limb onset ALS who were referred to the Department of Neurology of PUTH from 1 January, 2009 to 31 December, 2013. All patients, who were from mainland China, were diagnosed according to the Airlie House diagnostic criteria by neurologists from the Department of Neurology, PUTH (9). All selected patients had an insidious onset of weakness or muscle atrophy in the legs and gradually progressive leg motor dysfunction. The detailed demographic information and clinical data including the ALS Functional Rating Scale-Revised (ALSFRS-R) score were gathered from each patient during the first visit in our department. The clinical symptoms and signs, including but not limited to, skeletal muscle strength and tone, deep tendon reflexes, upper motor neuron (UMN) signs, including the palmomental reflex; jaw jerk; Hoffmann's and Rossolimo's sign;Babinski's sign; and lower motor neuron (LMN) signs, including muscle atrophy, fasciculation or fibrillation, were collected. All patients completed nerve conduction studies (NCS) and electromyography (EMG), magnetic resonance imaging (MRI) of the central nervous system (CNS), and serum autoantibody tests. We excluded alternative diagnoses such as spinal cord compression or spinal stenosis, spinal and bulbar muscular atrophy (SBMA), spinal muscular atrophy (SMA), primary lateral sclerosis (PLS), chronic inflammatory demyelinating polyneuropathy (CIDP) and multifocal motor neuropathy (MMN). The patients with a family history of ALS or an onset age under 18 were excluded from the study.

We also evaluated forced vital capacity (FVC) during the patient's first visit in PUTH. The FVC values were expressed as percentages of the predicted values (10). The FVC values <80% were considered abnormal, representing ventilation dysfunction (10). The patients with respiratory diseases that might affect the evaluation of FVC were excluded.

Each patient was given a telephone follow-up every 3 or 6 months until 31 December, 2020. At each follow-up, the ALSFRS-R was evaluated, and information on the patients' use of riluzole, non-invasive ventilation (NIV), and percutaneous endoscopic gastrostomy (PEG) tubes was collected by telephone. “Diagnostic delay” was defined as the time from symptom onset to a confirmed diagnosis of ALS made by a board-certified neurologist (3). “Use of riluzole” was defined as the treatment with riluzole (50 mg) two times a day for longer than 2 weeks (3). “Death or tracheotomy” was predefined as the primary outcome measure. “Survival time” was defined as the time from the onset of symptoms to either the date of the primary outcome measure or the end date of follow-up (31 December, 2020).

The defining characteristic of FLS was the duration from the lower limb onset to the second region significantly involved (SRSI), which was distinctly longer than that of typical lower limb onset ALS. The SRSI was defined as symptomatic upper limb weakness or dysarthria or dysphagia that led to the clinical manifestation, which emerged next to weakness in the lower limbs. If a patient with the lower limb onset ALS was found to have another region involved in the first visit in our department, the SRSI and the duration from onset to SRSI were recorded. If symptoms were still confined to lower limbs, the information on SRSI was acquired through a follow-up by telephone and recorded in the ALSFRS-R. The patients were excluded if they were unable to identify the exact time of SRSI or were lost to follow-up.

### Statistical Analysis

A Cox regression analysis was performed to understand which factor could impact survival. These factors included the gender; onset age; diagnostic delay; duration from lower limb onset to SRSI; ALSFRS-R score at the first visit in PUTH; and use of riluzole, NIV, and PEG. Then a cutoff point analysis was performed by maximally selected log–rank statistics using R software v. 3.4.2 (packages: maxstat) (11). The value of the duration in months from lower limbs to SRSI was analyzed as a continuous variable and each value of the duration was analyzed as a candidate cutoff value. Based on the maximum relative risk and minimum *p*-value, the cutoff value of the duration that optimally dichotomized patients' outcomes was selected to distinguish slowly progressive subtype of lower limb onset ALS from typical lower limb onset ALS.

We divided all patients into the slowly progressive subtype of the lower limb onset ALS and typical lower limb onset ALS groups based on the cutoff value. In the former group, the patients were identified as having FLS, who had significant bilateral lower extremity muscle weakness and had no hypertonia or clonus in lower limbs according to the clinical history combined with physical examination during the patients' first visit to our department. The differences in demographic and clinical features were compared between the patients with FLS and the patients with typical lower limb onset ALS by SPSS 18.0 software for Windows (SPSS Inc., Chicago, IL, USA). The quantitative data that were normally distributed were expressed as mean ± standard deviations (SDs), and quantitative data that were non-normally distributed were expressed as the medians and ranges (minimums, maximums) (12). The differences in categorical variables were analyzed by χ^2^ test or Fisher's exact test, and continuous variables were evaluated by an independent-sample *t*-test or the Mann–Whitney *U*-test depending on the parametric or non-parametric nature of the data (12). The differences in survival between the two groups were compared using Kaplan–Meier analysis and the log-rank test. *P* < 0.05 (two-sided) was considered statistically significant.

## Results

Among the 255 patients diagnosed with the lower limb onset ALS in the patient database of those with ALS, 196 patients met the inclusion criteria for the study. Among the 59 patients who were excluded, four patients were unable to identify the exact time of SRSI, and the remaining 55 patients were lost to follow-up.

A total of 196 patients with the lower limb onset ALS (109 men, 87 women, mean age 53.1 ± 11.2 years, range, 23–88 years) exhibited a mean onset age of 51.8 ± 11.2 years (range, 21–88 years), a median diagnostic delay of 12 (2, 78) months from the lower limb onset, and a mean ALSFRS-R score of 37.3 ± 6.5 (range, 17–47) at the first visit in PUTH. Regarding the side of onset, 82 patients (41.9%) had right lower limb onset, 75 patients (38.3%) had left lower limb onset, and the remaining 39 patients (19.9%) had simultaneous onset in both lower limbs. The median duration from the lower limb onset to SRSI was 8 (1, 60) months. The sites of SRSI included the unilateral upper limb (160 patients, 81.6%); both upper limbs simultaneously (22 patients, 11.2%), the bulbar region (10 patients, 5.1%); and the upper limb and bulbar region at almost the same time (four patients, 2.0%). A total of 188 patients (95.9%) suffered primary outcome events, including 12 patients treated with a tracheotomy, by 31 December, 2020. The median survival time was 34 (7, 151) months. Riluzole, NIV, and PEG were used by 65 (33.2%), 23 (11.7%), and 1 (0.5%) patients, respectively.

### Cutoff Value of the Duration From Lower Limb Onset to SRSI

**Figure 1:**
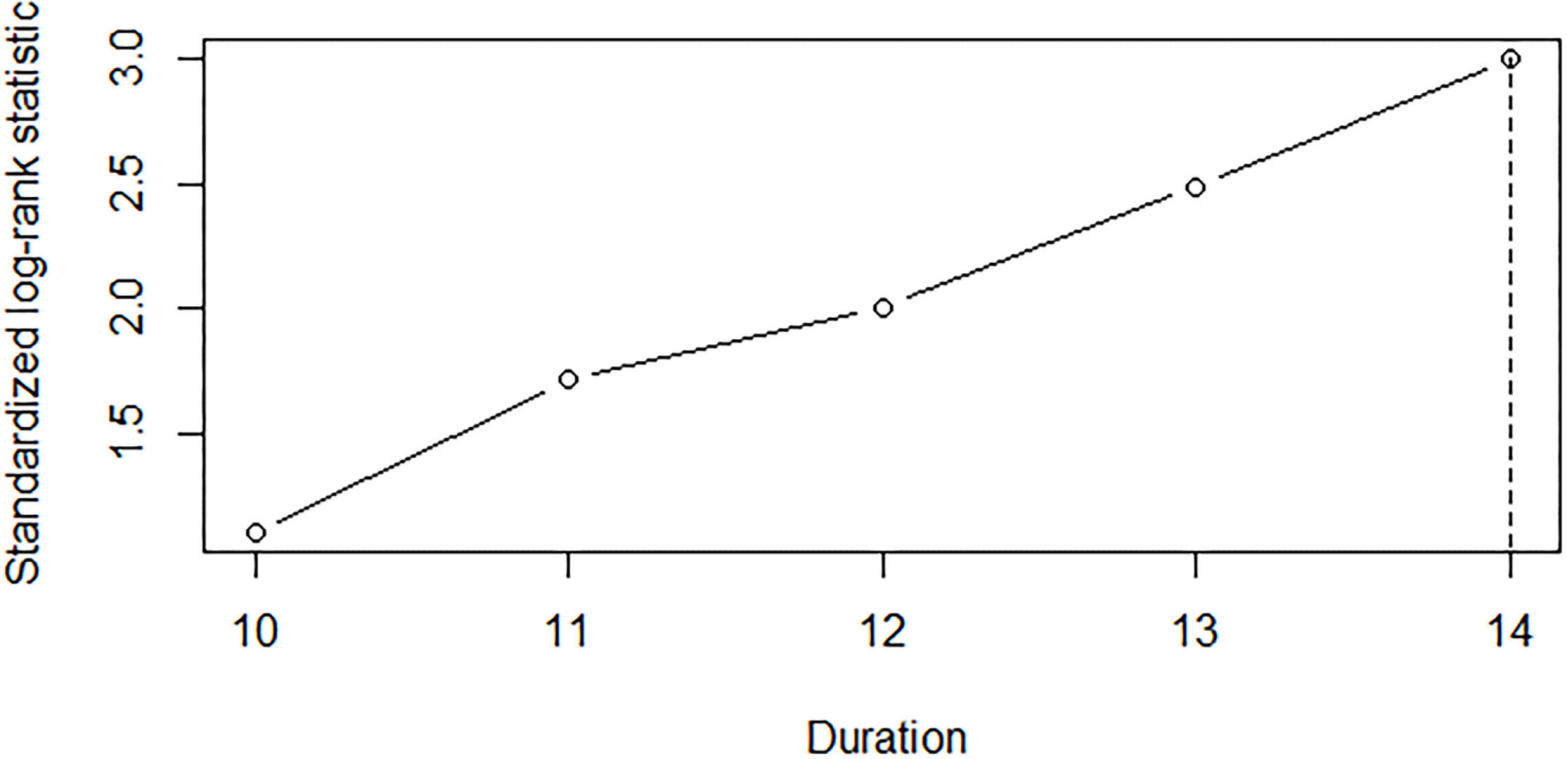
A cutoff point analysis of the duration from the lower limb onset to SRSI in 196 lower limb onset patients with ALS. Each value of the duration in months from the lower limb onset to SRSI was analyzed as a candidate cutoff value by maximally selected log–rank statistics. The duration of 14 months was selected, which optimally dichotomized patients' survival. SRSI, second region significantly involved; ALS, amyotrophic lateral sclerosis.

The Cox regression analysis showed that the duration from lower limb onset to SRSI (*p* < 0.001); onset age (*p* < 0.001); diagnostic delay (*p* < 0.001); ALSFRS-R score at the first visit in PUTH (*p* = 0.003); and use of riluzole (*p* = 0.037) and NIV (*p* = 0.017) had significant impacts on survival (Table 1). Two other factors, including gender (*p* = 0.256) or use of PEG (*p* = 0.302) had no significant impact on survival. A cutoff point analysis performed by maximally selected log-rank statistics indicated that the cutoff value of the duration from lower limb onset to SRSI was 14 months (Figure 1). According to the cutoff value, slowly progressive subtype of lower limb onset ALS was defined as patients with ALS who had a duration more than or equal to 14 months from lower limb onset to SRSI; in contrast, typical patients', with lower limb onset ALS, duration was <14 months.

**Table 1:** Multivariate Cox regression analysis of the impact of factors on survival in patients with lower limb onset ALS.

**Variable**	**HR**	**95% CI**	***p***
**Gender**			
Women	1.000		
Men	1.191	0.881–1.609	0.256
Onset age (years)	1.034	1.019–1.049	<0.001
Diagnostic delay (months)	0.960	0.939–0.982	<0.001
Duration (months)	0.895	0.866–0.926	<0.001
ALSFRS-R	0.957	0.929–0.985	0.003
**Use of riluzole**			
No	1.000		
Yes	0.713	0.519–0.980	0.037
**Use of NIV**			
No	1.000		
Yes	0.553	0.340–0.900	0.017
**Use of PEG**			
No	1.000		
Yes	2.933	0.381–22.608	0.302

*ALS, amyotrophic lateral sclerosis; HR, hazard ratio; CI, confidence interval; Duration, duration from lower limb onset to second significant region involvement; ALSFRS-R, ALS Functional Rating Scale-Revised; NIV, non-invasive ventilation; PEG, percutaneous endoscopic gastrostomy*.

### Comparison of Demographic and Clinical Features

Based on a duration <14 months from lower limb onset to SRSI, 157 patients (80.1%) were identified as having typical lower limb onset ALS [male to female ratio 1.3: 1, mean onset age 52.9 ± 11.2 years (range, 21–88 years), median diagnostic delay 11 (2, 43) months, mean ALSFRS-R score 36.6 ± 6.4, simultaneous onset in both lower limbs in 28 patients (17.8%)]. Among the 39 patients with a duration more than or equal to 14 months, 29 patients (14.8%) were classified as having FLS [male to female ratio 1.4: 1, mean onset age 49.6 ± 9.4 years (range, 26–66 years), median diagnostic delay 25 (5, 60) months, mean ALSFRS-R score 40.1 ± 5.3, simultaneous onset in both lower limbs in 8 patients (27.6%)], who had significant bilateral lower extremity muscle weakness and had no hypertonia or clonus in lower limbs according to the records of physical examination during the patients' first visit in our department. The other 10 patients (5.1%) with slowly progressive subtype of lower limb onset ALS were excluded from the FLS group, including two patients having unilateral lower extremity weakness and eight patients having hypertonia or clonus in lower limbs. The demographic and clinical characteristics of the patients with FLS and typical patients with lower limb onset ALS at the first visit in PUTH are shown in Table 2.

**Table 2:** Baseline characteristics of different groups of patients with lower limb onset ALS.

**Groups**	**Gender ratio, male:female**	**Onset age (years)**	**Diagnostic delay (months)**	**Bilateral LL Onset, *n* (%)**	**ALSFRS-*R* score**	**Babinski's sign, *n* (%)**	**Brisk deep tendon reflex of LL, *n* (%)**	**Decreased deep tendon reflex of LL, *n* (%)**
**FLS** (*n* = 29)	1.4:1	49.6 ± 9.4	25(5,60)	8(27.6)	40.1 ± 5.3	11(37.9)	10(34.5)	15(51.7)
**Typical LL onset**
**ALS (*****n*** **=** **157)**	1.3:1	52.9 ± 11.2	11(2,43)*	28(17.8)	36.6 ± 6.4*	69(43.9)	70(44.6)	60(38.2)
**Statistics**	0.037^†^	1.485^‡^	5.047^§^	1.491^†^	2.723^‡^	0.362^†^	1.019^†^	1.856^†^
*p*	0.847	0.139	<0.001	0.222	0.007	0.548	0.313	0.173

*Values are presented as n (%), mean ± SD or median (minimum, maximum)*.

*p-value for the contrast of variables between patients with FLS and patients with typical lower limb onset ALS; p < 0.05, as compared with patients with FLS. χ^2^-values;

*^‡^t-values*;

*^§^Z-values. ALS, amyotrophic lateral sclerosis; LL, lower limb; ALSFRS-R, ALS Functional Rating Scale-Revised; FLS, flail leg syndrome; SD, standard deviation*.

In clinical signs, the palmomental reflex or jaw jerk, Hoffmann's or Rossolimo's sign, Babinski's sign, brisk upper limb deep tendon reflex, decreased or absent upper limb deep tendon reflex, brisk lower limb deep tendon reflex and decreased or absent lower limb deep tendon reflex were found in 5, 14, 11, 9, 15, 10, and 15 patients in the FLS group, and in 55, 85, 69, 62, 70, 70, and 60 patients in the typical lower limb onset ALS group, respectively.

The diagnostic delay was longer and the ALSFRS-R score at the first visit in PUTH was higher in the FLS group than in the typical lower limb onset ALS group (*p* < 0.001, *p* = 0.007). No significant differences in the gender ratio, onset age, the proportion of simultaneous onset in both lower limbs or the proportion of those above signs were found between groups.

Combined with the data of patients' first visit in our department and follow-up, the median duration from lower limb onset to SRSI was 24 (14, 60) months for patients with FLS and 6 (1, 13) months for typical patients with lower limb onset ALS. Motor dysfunction progressed to SRSI significantly later in the FLS group than in the typical lower limb onset ALS group (*p* < 0.001). The unilateral upper limb; both upper limbs simultaneously; the bulbar region; and the upper limb and bulbar region at almost the same time were second significantly involved in 123, 22, 8, and 4 patients in the typical lower limb onset ALS group, respectively. Except for two patients whose sites of SRSI were the bulbar region, the remaining 27 patients had the unilateral upper limb involvement following lower limb onset in the FLS group. As the sites of SRSI, both upper limbs simultaneously were less frequent in the FLS group than in the typical lower limb onset ALS group (*p* = 0.028). No significant differences in the other three sites of SRSI were found between groups.

Riluzole, NIV, and PEG were used by nine (31.0%), two (6.9%), and 0 patients in the FLS group and by 53 (33.8%), 21 (13.4%), and 1 (0.6%) patients in the typical lower limb onset ALS group, respectively. There were no significant differences in the percentages of patients using riluzole, NIV or PEG in either group.

Seventy-nine patients (40.3%) completed FVC examination at the first visit in our department, including 16 patients in the FLS group [10 males, 6 females, mean onset age 48.8 ± 8.7 years, diagnostic delay 23.5 (5, 60) months, mean ALSFRS-R score 41.2 ± 4.6, mean FVC value 90.4 ± 14.5% (range, 54–111%) of the predicted value] and 63 patients in the typical lower limb onset ALS group [38 males, 25 females, mean onset age 53.0 ± 10.9 years, diagnostic delay 11 (2, 42) months, mean ALSFRS-R score 36.5 ± 6.3, mean FVC value 85.2 ± 16.4% (range, 47–115%) of the predicted value] (Table 3). Two patients with FLS (12.5%) and 27 typical patients with lower limb onset ALS (42.9%) had an FVC of <80%. The incidence of FVC being <80% was lower; the ALSFRS-R score at the first visit in our department was higher and the diagnostic delay was longer in the FLS group than in the typical lower limb onset ALS group (*p* = 0.024; *p* = 0.006; *p* = 0.001). There were no significant differences in FVC value, gender ratio or onset age in either group. The evaluation of FVC was not finished in the remaining 117 patients owing to patients' intolerance of FVC examination or the long appointment time.

**Table 3:** FVC of 79 patients with lower limb onset ALS.

**Groups**	**Gender ratio, male:female**	**Onset age** **(years)**	**Diagnostic delay (months)**	**ALSFRS-R score**	**FVC of predicted, (%)**	**FVC <80%, *n* (%)**
**FLS** (*n* =16)	1.7:1	48.8 ± 8.7	23.5 (5,60)	41.2 ± 4.6	90.4 ± 14.5	2 (12.5)
**Typical LL onset**
**ALS** (*n* = 63)	1.5:1	53.0 ± 10.9	11 (2,42)*	36.5 ± 6.3*	85.2 ± 16.4	27 (42.9)*
**Statistics**	0.025^†^	1.450^‡^	3.341^§^	2.829^‡^	1.164^‡^	5.061^†^
***p***	0.873	0.151	0.001	0.006	0.248	0.024

*Values are presented as n (%), mean ± SD or median (minimum, maximum)*.

*p-value for the contrast of variables between patients with FLS and typical patients with lower limb onset ALS; p < 0.05, as compared with patients with FLS. χ^2^-values;

*^‡^t-values*;

*^§^Z-values. FVC, forced vital capacity; ALS, amyotrophic lateral sclerosis; ALSFRS-R, ALS Functional Rating Scale-Revised; FLS, flail leg syndrome; LL, lower limb; SD, standard deviation*.

### Survival Analysis

**Figure 2:**
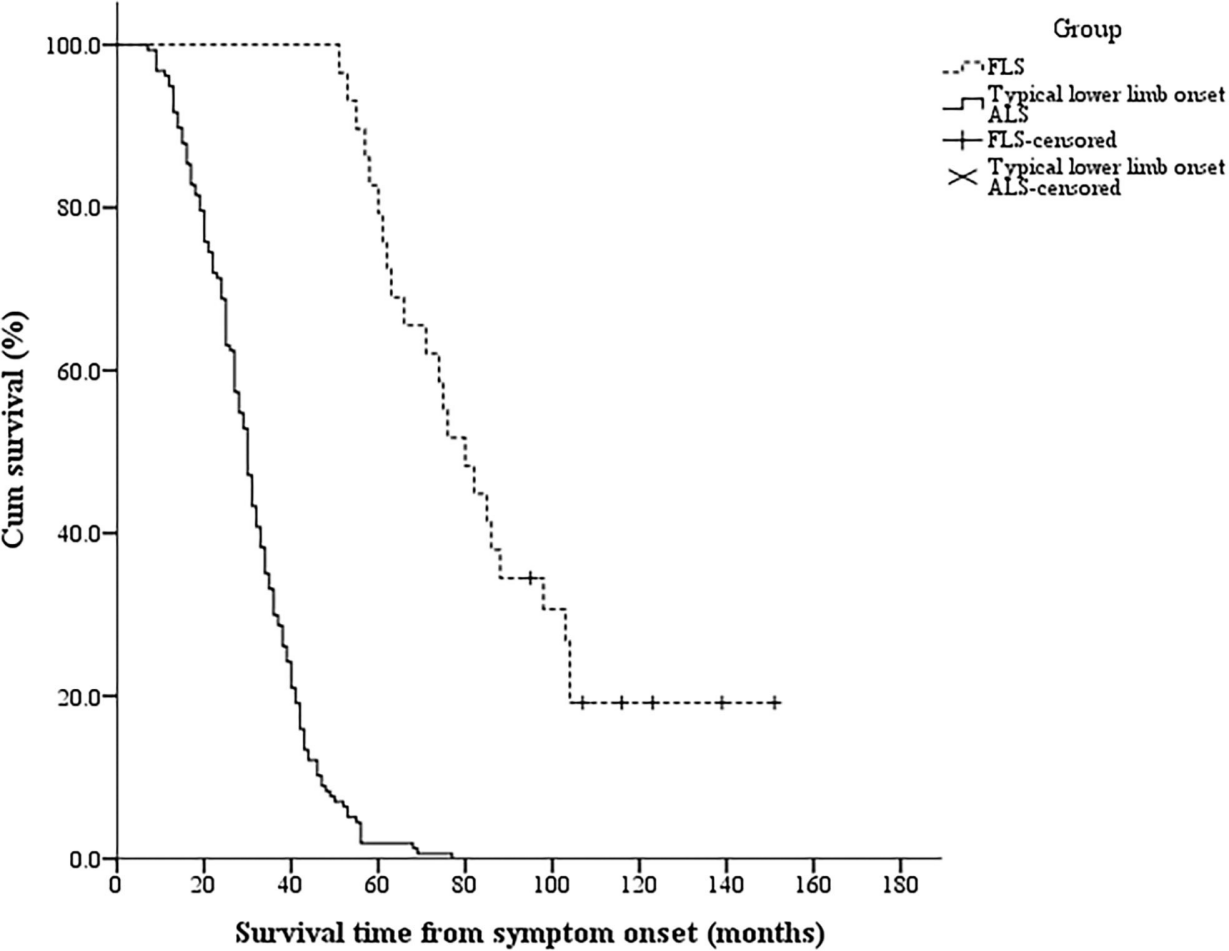
The Kaplan–Meier survival curves of different phenotypes in 186 patients with lower limb onset ALS. Twenty-nine patients were classified as having FLS and 157 patients were classified as having typical lower limb onset ALS according to the cutoff duration of 14 months from the lower limb onset to SRSI and clinical features. The log–rank test indicated that the median survival time was significantly longer in the FLS group than in the typical lower limb onset ALS group (*p* < 0.001). ALS, amyotrophic lateral sclerosis; SRSI, the second region significantly involved; FLS, flail leg syndrome.

There were six patients with FLS (20.7%, three men) and no patients with typical lower limb onset ALS surviving until the end of the follow-up period. The 5-year survival rate was much higher in patients with FLS (79.3%) than in patients with typical lower limb onset ALS (1.9%). The median survival time of patients with FLS [80 (51, 151) months] was significantly longer than that of patients with typical lower limb onset ALS [30 (7, 77) months] (log–rank = 85.330, *p* < 0.001). The Kaplan–Meier curves are shown in Figure 2.

No significant differences were noted in the onset age, diagnostic delay, side of onset proportion, the ALSFRS-R score at the first visit in our department, duration from the lower limb onset to SRSI or survival time between men and women in the FLS group.

**Figure 3:**
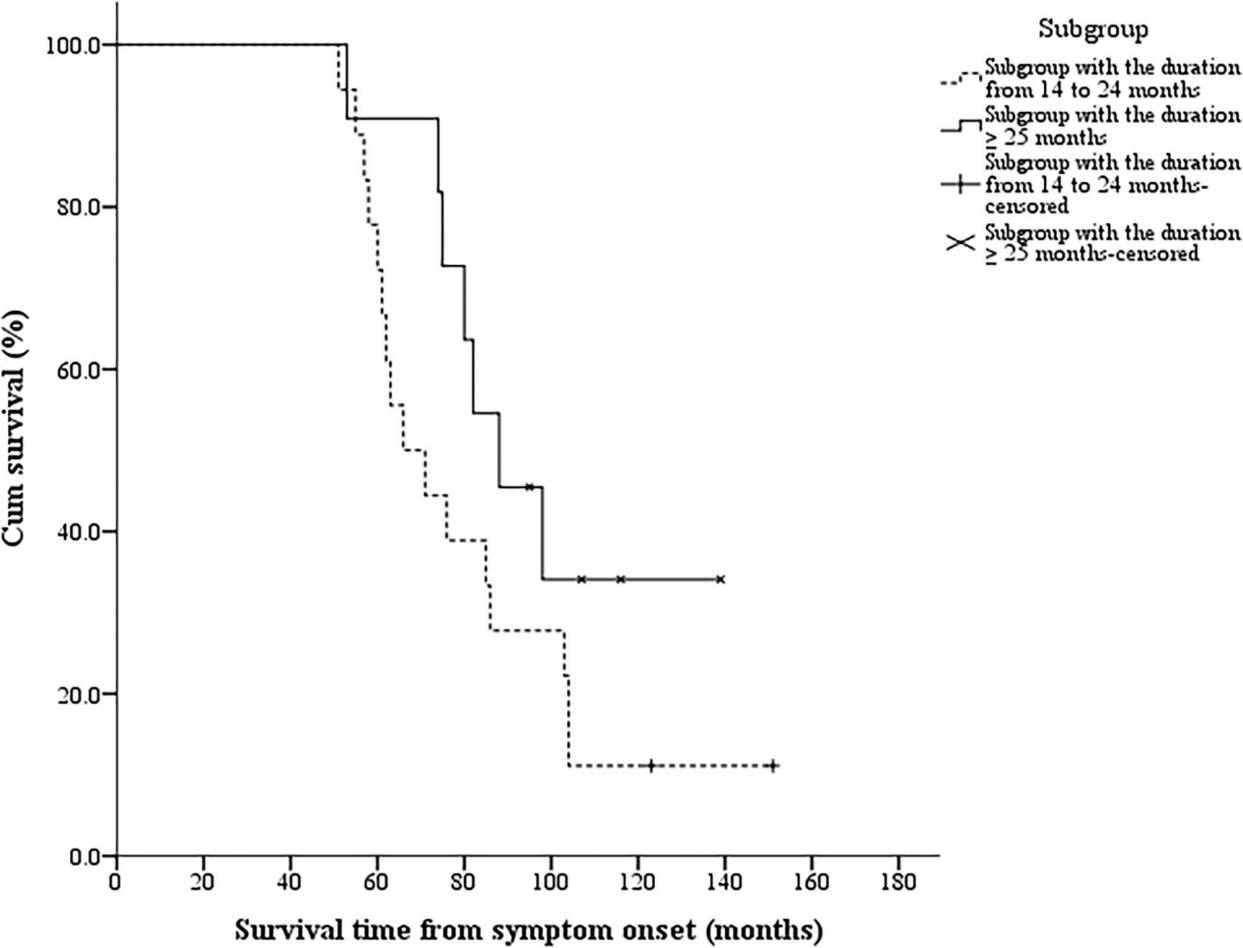
The Kaplan–Meier survival curves of 29 patients with FLS in different subgroups. Twenty-nine patients with FLS were divided into two subgroups according to the duration of 24 months from the lower limb onset to SRSI: 18 patients had a duration from 14 to 24 months, and 11 patients had a duration more than or equal to 25 months. The log–rank test showed no significant differences in the median survival time between the two subgroups (*p* = 0.133). FLS, flail leg syndrome; SRSI, the second region significantly involved.

Four patients (2.5%) in the typical lower limb onset ALS group had a duration from the lower limb onset to SRSI of 13 months (more than 12 months), and their survival time was 38, 46, 48, and 69 months, respectively. All 29 patients with FLS were divided into two subgroups according to the duration of 24 months from the lower limb onset to SRSI: 18 patients had a duration from 14 to 24 months, and 11 patients had a duration of more than or equal to 25 months. Two patients in the former subgroup and four patients in the latter subgroup survived until the end of the follow-up period, and the median survival time was 68.5 (51, 151) months and 88 (53, 139) months, respectively. The log–rank test showed no significant differences in the median survival time between the two subgroups (log–rank = 2.255, *p* = 0.133). The Kaplan–Meier curves of the two subgroups are shown in Figure 3.

## Discussion

Flail leg syndrome, which is also called leg amyotrophic diplegia (LAD), is a rare regional variant of ALS that is characterized by slower progression and better prognosis than typical lower limb onset ALS (4–7). It is difficult to distinguish FLS from the typical lower limb onset ALS, and the existing criteria are not uniform. In this study, the correlation between the duration from the lower limb onset to SRSI and survival was analyzed in patients with the lower limb onset ALS. Based on a cutoff point analysis, a cutoff duration of 14 months that optimally dichotomized patients' survival was obtained to distinguish FLS from the typical lower limb onset ALS. However, besides slow progression, FLS should also have the flail leg phenotype, such as the progressive distal onset of weakness and wasting in the lower limbs, but without hypertonia or clonus (4–7). Patients with wasting and weakness beginning proximally in the legs without distal involvement at presentation are excluded from FLS (4–8). In our study, patients who had a duration from the lower limb onset to SRSI ≥14 months and had significant bilateral lower extremity muscle weakness without hypertonia or clonus in lower limbs according to the records of physical examination during the patients' first visit in our department were identified as having FLS, whereas patients with a duration <14 months were classified as having the typical lower limb onset ALS. Of the 39 patients, 10 patients who had hypertonia or clonus in lower limbs or unilateral lower extremity weakness with a duration from the lower limb onset to SRSI ≥14 months were excluded from FLS. Although these patients had slow disease progression, they did not have the phenotype of flail leg and could only be classified as atypical lower limb onset ALS. So, patients with lower limb onset ALS, who had a duration from lower limb onset to SRSI <14 months or had hypertonia or clonus in lower limbs or had no distal lower limb involvement at presentation, were excluded from FLS.

The time of 14 months, which was defined as the cutoff duration to identify FLS based on statistical analysis in the present study, is between 12 and 24 months, which was chosen mainly according to clinical experience in two previous studies. Only 4 of 157 (2.5%) patients with the typical lower limb onset had a duration from the lower limb onset to SRSI between 12 and 14 months, and three of the four patients had a short survival time ≤4 years. These patients, who were diagnosed with the typical lower limb onset ALS according to the present cutoff value, might be identified as having FLS based on the cutoff duration of 12 months proposed by the UK and Melbourne study but had a worse prognosis (5). The cutoff duration of 12 months might slightly reduce the specificity of FLS diagnosis. Eighteen of 29 patients with FLS (62.1%) had a duration from 14 to 24 months, and their median survival time was significantly longer than that of patients with the typical lower limb onset ALS and was not significantly different from that of the remaining 13 patients with FLS with a duration more than 24 months. These patients, who were diagnosed with FLS according to the present cutoff value, would be identified as having typical lower limb onset ALS based on the cutoff duration of 24 months proposed by the US and major academic center study but had a better prognosis (6). A considerable number of patients with FLS might be diagnosed as having the typical lower limb onset ALS using the cutoff duration of 24 months, and the sensitivity of FLS diagnosis might be notably reduced. To date, there is no gold standard for the diagnosis of FLS, so it is difficult to accurately evaluate the sensitivity and specificity of the cutoff duration of 12, 14, or 24 months. Considering that our choice was based on a statistical analysis of clinical data, the cutoff duration of 14 months may be better than the other two cutoff values. However, it should be recognized that the cutoff duration may vary among different populations because of the impact of ethnic and regional differences, as well as the sample size, on the research results.

As a rare subtype, FLS accounted for 2.5 to 13.0% of patients with ALS and had a mean onset age between 55 and 65 years, similar to classic ALS in the previous reports (4–8). The FLS was characterized by a male predominance with a male to female ratio of between 1 and 7 to 1 and a better prognosis than classic ALS with a mean survival of 75.9 to 87 months and a 5-year survival rate of 63.9–76.9% (4–7). Both diagnostic delay and the median time from onset to NIV use were longer in FLS than in classic ALS (4, 5).

The demographic and clinical characteristics of patients with FLS in the present study were described and compared with those in the previous reports. The FLS accounted for 14.8% of the lower limb onset ALS and had a male to female ratio of 1.4:1, similar to the studies of Wijesekera et al. (5) and Kornitzer et al. (13). The mean onset age of patients with FLS was 49.6 ± 9.4 years and was about 5–15 years younger than that in previous studies. The onset age of Chinese patients with ALS was approximately a decade younger than that of European patients, and the differences in genetic background and lifestyle might lead to different disease susceptibility (3). The Cox regression analysis showed that a younger onset age, a longer diagnostic delay, a higher ALSFRS-R score, or the use of riluzole or NIV were associated with the trend of benign survival, which has been found in previous studies (1, 3, 5). The duration from the lower limb onset to SRSI, the diagnostic delay, and the survival time were longer and the ALSFRS-R score and the 5-year survival rate were higher in FLS than in the typical lower limb onset ALS, which are consistent with the features of comparatively isolated lumbosacral symptoms, slower progression to other CNS regions, and a comparatively benign prognosis of FLS (4–7). Patients with typical lower limb onset ALS were more frequent to have both upper limbs simultaneously as the sites of SRSI, which might be a predictor of typical lower limb onset ALS. Also, ALS starts focally in a CNS region and usually spreads to adjacent regions. Gromicho et al. (14) found that the contiguous spreading was the leading progression pattern in ALS, and the regional progression of LMN degeneration was most likely contiguous. The horizontal and the vertical directions were the major spread directions in lower limb onset ALS (15). Simultaneous bilateral upper limb involvement means that the bilateral pyramidal tracts or anterior horns of the cervical spinal cord are involved at almost the same time, which indicates a relatively rapid progression. The bulbar region was found as the site of SRSI in both FLS and typical lower limb onset ALS groups, but the incidence was low. Cross-regional spread is less common in ALS, and its mechanism is unclear (15). In the US and major academic center study, patients were excluded from FLS if they had brisk or normal deep tendon reflexes of the legs or Babinski's sign at 2 years or at the first evaluation if conducted more than 2 years after onset (6). In the studies of Wijesekera et al. (5), Chiò et al. (8), and Kornitzer et al. (13), although FLS was characterized by decreased or absent deep tendon reflexes in lower limbs, some patients with FLS could have brisk lower limb deep tendon reflexes or Babinski's sign at some point during the disease. The proportion of brisk lower limb deep tendon reflexes or Babinski's sign in patients with FLS remains unclear. In our study, in the clinical signs recorded during the patients' first visit in PUTH, including limb deep tendon reflexes, the palmomental reflex or jaw jerk, Hoffmann's or Rossolimo's sign, and Babinski's sign, there were no significant differences between FLS and typical lower limb onset ALS. Babinski's sign, brisk lower limb deep tendon reflexes, and decreased or absent lower limb deep tendon reflexes were found in 37.9, 34.5, and 51.7% patients with FLS, respectively. A potential explanation for these divergences is that our patient inclusion criteria and study methods are different from those in previous studies. On the basis of statistical analysis, we found that patients with ALS with a duration more than or equal to 14 months from the lower limb onset to SRSI were more likely to be patients with FLS, which was the main difference from the previous studies. Patients with hypertonia or clonus were excluded from FLS, but patients with other UMN signs, including Babinski's sign or brisk lower limb deep tendon reflexes, were not excluded from FLS. We believe that FLS, as a subtype of ALS with features of upper and LMN degeneration, can have UMN signs in the legs, which is not the key differential point between FLS and typical lower limb onset ALS. Another possible reason for no significant differences in the signs between the two groups may be the small sample size of patients with FLS.

The present study showed that the incidence of FVC being <80% was lower in FLS than in the typical lower limb onset ALS, which coincides with the later NIV use and the lower rate of loss of vital capacity in FLS in previous studies (5, 13). The most common SRSI in both FLS and the typical lower limb onset ALS is the cervical spinal cord, whose third to fifth segments innervate the diaphragm. Motor neuron degeneration in FLS is relatively isolated to the lumbosacral spinal cord for a longer period of time, which provides a potential explanation for the later damage of the diaphragm and slower respiratory function involved in FLS. In our study, the proportion of patients using NIV was not significantly different between patients with FLS and patients with typical lower limb onset ALS. Some patients who had an FVC of <80% did not use NIV because of expense or intolerance of NIV, while others with normal FVC began to use NIV to reduce the subjective symptoms of dyspnea. The difference in the mean FVC value was not significant between the two groups. Two potential explanations could account for the result. A longer diagnostic delay in FLS indicated that FVC was assessed notably later from symptom onset in FLS than in typical lower limb onset ALS, which could decrease the FVC value of patients with FLS. The respiratory function of patients with ALS usually declines with the course of the disease (2, 13). Another explanation may be that the sample size was small and only 79 patients (40.3%) completed the FVC examination.

The time of disease progression is very important for the diagnosis of FLS. If a patient has a typical FLS phenotype, but muscle weakness and atrophy rapidly progress from lower limbs to other regions in a short time, the diagnosis is more likely to be typical ALS than FLS. A time that could feasibly differentiate survival between FLS and patients with the typical lower limb onset ALS to the greatest extent would be ideal in clinical application. We found that a cutoff duration of 14 months optimally dichotomized patients' survival, which might be a crucial feature to distinguish FLS from the typical lower limb onset ALS in the Chinese population. FLS was characterized by slower progression, fewer bilateral upper limbs simultaneously involved, less and later respiratory dysfunction, and a more benign prognosis than the typical lower limb onset ALS. These findings need further evaluation by multicenter studies.

Identifying FLS may help to carry out a meaningful prognostic consultation for patients with the lower limb onset ALS, especially when symptoms progress beyond the lumbosacral region. Choosing the proper treatment and care for patients will also be improved. In clinical trials including patients with FLS or other regional variants of ALS, researchers should consider stratifying the patients to reduce the variability in response to therapy owing to the differences in the rate of progression (4).

## Data Availability Statement

The raw data supporting the conclusions of this article will be made available by the authors, without undue reservation.

## Ethics Statement

The studies involving human participants were reviewed and approved by the Ethical Committee of Peking University Third Hospital. The patients/participants provided their written informed consent to participate in this study.

## Author Contributions

Material preparation, data analysis, and draft manuscript were executed by HZ. LC and JT collected the data. DF revised the manuscript for intellectual content. All authors contributed to the study's conception and design and approved the final version of the manuscript.

## Funding

This study was supported by the National Natural Science Foundation of China (81873784 and 82071426) and the Clinical Cohort Construction Program of Peking University Third Hospital (BYSYDL2019002).

## Conflict of Interest

The authors declare that the research was conducted in the absence of any commercial or financial relationships that could be construed as a potential conflict of interest.

## Publisher's Note

All claims expressed in this article are solely those of the authors and do not necessarily represent those of their affiliated organizations, or those of the publisher, the editors and the reviewers. Any product that may be evaluated in this article, or claim that may be made by its manufacturer, is not guaranteed or endorsed by the publisher.

## Abbreviations

ALS: amyotrophic lateral sclerosis
ALSFRS-R: Revised ALS Functional Rating Scale
CIDP: chronic inflammatory demyelinating polyneuropathy
CNS: central nervous system
EMG: electromyography
FLS: flail leg syndrome
FVC: forced vital capacity
LAD: leg amyotrophic diplegia
LMN: lower motor neuron
MMN: multifocal motor neuropathy
MRI: magnetic resonance imaging
NCS: nerve conduction studies
NIV: non-invasive ventilation
PEG: percutaneous endoscopic gastrostomy
PLS: primary lateral sclerosis
PUTH: Peking University Third Hospital
SBMA: spinal and bulbar muscular atrophy
SMA: spinal muscular atrophy
SRSI: second region significantly involved
UMN: upper motor neuron.
